# Stereotactic Radiosurgery (SRS) Induced Higher-Grade Transformation of a Benign Meningioma into Atypical Meningioma

**DOI:** 10.1155/2022/4478561

**Published:** 2022-02-23

**Authors:** Ali Basalamah, Mohammed Al-bolbol, Osman Ahmed, Nagoud Ali, Sabah Al-Rashed

**Affiliations:** ^1^Department of Neurosurgery, King Saud University Medical City/King Khalid University Hospital, Riyadh, Saudi Arabia; ^2^Department of Neurosurgery, Prince Sultan Military Medical City, Riyadh, Saudi Arabia; ^3^Department of Pathology, Prince Sultan Military Medical City, Riyadh, Saudi Arabia

## Abstract

**Background:**

Stereotactic radiosurgery (SRS) is a widely used treatment modality for the management of meningioma. Whether used as a primary, adjuvant, or salvage procedure, SRS is a safe, less invasive, and effective modality of treatment as microsurgery. The transformation of a meningioma following radiosurgery raises a concern, and our current understanding about it is extremely limited. Only a few case reports have described meningioma dedifferentiation after SRS to a higher grade. Moreover, a relatively small number of cases have been reported in large retrospective studies with little elaboration. *Case Description*. We report a detailed case description of a 41-year-old man with progressive meningioma enlargement and rapid grade progression after SRS, which was histopathologically confirmed before and after SRS. We discussed the clinical presentation, radiological/histopathological features, and outcome. We also reviewed previous studies that reported the outcome and follow-up of patients diagnosed with grade I meningioma histopathologically or presumed with benign meningioma by radiological features who underwent primary or adjuvant radiosurgery.

**Conclusion:**

The risk of progression after SRS is low, and the risk of higher-grade transformation after SRS is trivial. The early timing for recurrence and field-related radiation may favor a relationship between SRS and higher-grade transformation (causality) although transformation as a part of the natural history of the disease cannot be fully excluded. Tumor progression (treatment failure) after SRS may demonstrate a transformation, and careful, close, and long follow-up is highly recommended. Also, acknowledging that there is a low risk of early and delayed complications and a trivial risk of transformation should not preclude its use as SRS affords a high level of safety and efficiency.

## 1. Introduction

Meningioma is a common intracranial tumor accounting for 15-20% and up to 1/3 of all intracranial tumors in several other series [1, 2]. The World Health Organization (WHO) classified meningiomas as benign (grade I), atypical (grade II), and malignant (grade III). Benign meningiomas account for up to 90% of meningiomas, and it has an overall less than 2% risk of malignant progression [3].

Since the early description of Simpson's grading in 1957, gross total resection remained the mainstay and the primary goal of management for meningioma [2, 4]. Skull base and posterior fossa as compared to supratentorial meningiomas pose a high surgical challenge as they are nearby a crucial structure precluding complete removal [2, 5]. As a result, there is an increasing trend advocating for subtotal resection to decrease the risk of injury to the surrounding structures. Postsurgical intervention, selected patients may go for further external beam radiation therapy or SRS directed to the residual tumor to prevent recurrence [6].

Higher-grade transformation of a tumor can be defined as a histological change to a higher grade of a previously proven benign tumor histopathologically or presumed based on neuroimaging typical features of benign meningioma [7]. Also, metabolic studies can be used to indicate a transformation [8].

SRS is currently a widely applied procedure for meningioma management principally for skull base tumors [2]. Whether used as a primary, adjuvant, or salvage, SRS is a safe, less invasive, and effective modality of treatment as microsurgery [3, 9]. The disease control rate for SRS ranges from 87 to 100% and 67 to 100% at 5 and 10 years, respectively, with a 92.3% rate of overall symptom control. The estimated progression-free survival rate is ranging from 78 to 98.9% at 5 years and 53.1 to 97.2% at 10 years [9].

The transformation for a meningioma following radiosurgery raises a concern, and our current understanding about it is highly limited [10, 11]. Few case reports exist describing meningioma dedifferentiation after SRS to a higher grade. Moreover, a small number of cases were reported in large retrospective studies with relatively little elaboration. Hereby, we report a detailed case description of a 41-year-old man who had a progressive enlarging meningioma and transformation rapidly after SRS with a histopathological confirmation before and after SRS.

## 2. Case Presentation

**Figure 1:**
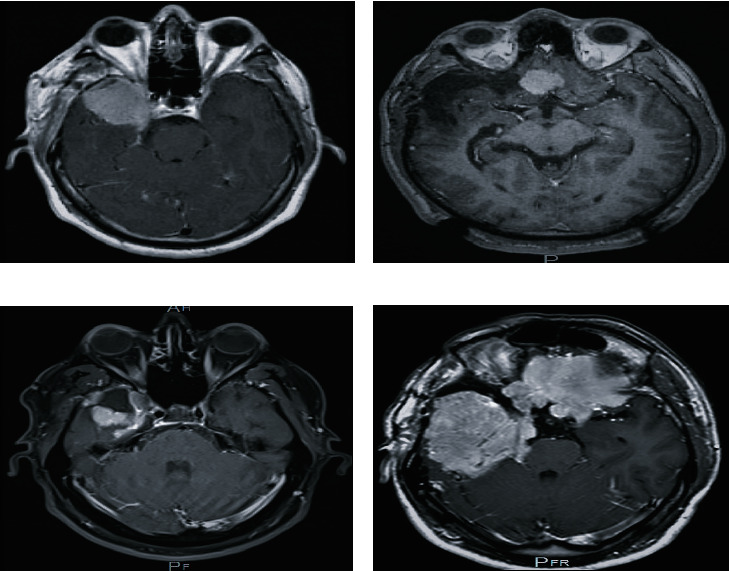
Neuroimaging–MRI ((a–d) with contrast enhancement) shows (a) sphenoid wing meningioma. (b) Frontobasal tumor projecting towards the right side (pre-SRS). (c) Tumor residual occupying the posterior aspect of the sphenoid wing and parasellar region (post-SRS). (d) Tumor occupies all the right middle cranial fossa extending to the cavernous sinuses, sella, and suprasellar regions as well as right prepontine and CP angle. It is also spread to the ipsilateral right superior orbital fissure and a lesser extent optic canal. The second component within the left anterior cranial fossa crossing the midline to the contralateral side and posteriorly to the left sphenoid ridge (post-SRS).

**Figure 2:**
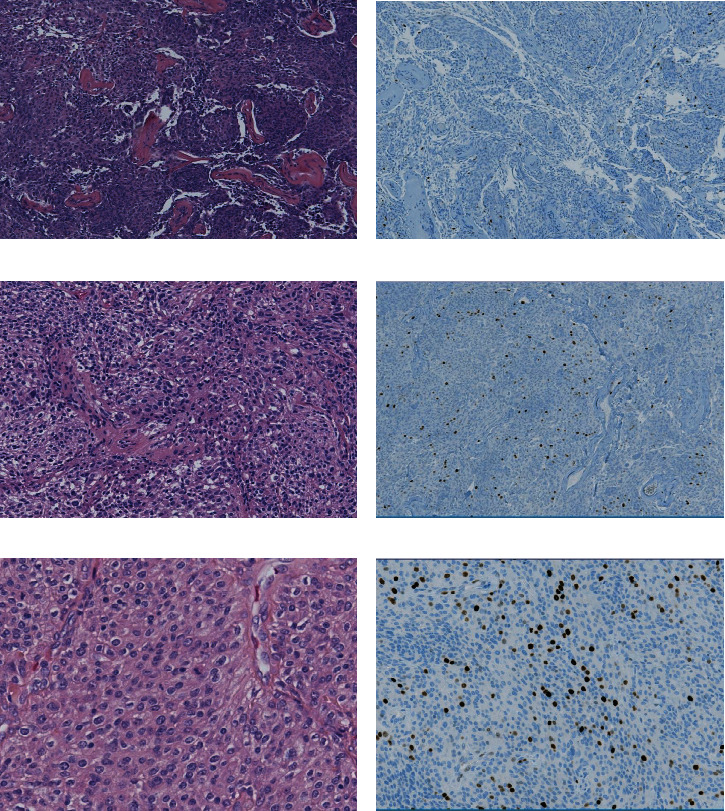
Histopathology. (2011) H&E-stained slide shows meningothelial meningioma, WHO grade I (a). Ki67 index (b). (2015) H&E-stained slide shows recurrent WHO grade I meningioma (c). Ki67 index (d). (2018) H&E-stained slide shows recurrent meningioma with atypical features and increased mitoses, WHO grade II (e). Ki67 index elevated (f).

A 41-year-old diabetic man presented in 2010 with moderate intermittent bifrontal dull headaches associated with visual disturbance and lacrimation. His initial vital signs, higher mental function, neurological and physical examinations, and laboratory results were unremarkable. Initial magnetic resonance imaging (MRI) revealed a large, well-defined extra-axial mass lesion, suggesting right sphenoid wing meningioma (Figure 1(a)). He underwent two stages surgical interventions. In January 2010, preoperative embolization was done, followed by partial excision of the tumor. Full resection was precluded due to material-related tissue reaction, solid tumor consistency, and patient's transient intraoperative instability. Histopathologically, it was reported as meningothelial meningioma (WHO I) (Figures 2(a) and 2(b)). The second-stage surgery was repeated in January 2011. A frontotemporal craniotomy and near-total excision of the tumor was achieved. A small residual tumor remained attached along the right optic nerve and chiasm. Histopathologically, meningothelial meningioma was confirmed. He has been following in our neurosurgical clinic since 2010. A frontobasal tumor projecting towards the right side was found during follow-up imaging in 2013 (Figure 1(b)). In 2014, a new MRI revealed an increased size of the residual tumor along the optic nerve and above the chiasm, as well as progression of the frontobasal component. He was treated with SRS in late 2014. In 2015, he underwent total surgical resection for the frontobasal component due to poor local control of the tumor. Histopathologically, meningothelial meningioma (WHO I) was reported (Figures 2(c) and 2(d)). In 2016, MRI showed a significant interval increase in the size of the residual tumor in the right middle fossa and right parasellar regions. In February 2018, MRI revealed a tumor occupying the entire right middle cranial fossa extending to the cavernous sinuses, sella, and suprasellar regions, as well as the right prepontine and CP angle. It is also extending to the ipsilateral right superior orbital fissure and the optic canal to a lesser extent. The second component was seen within the left anterior cranial fossa crossing the midline to the contralateral side and posteriorly to the left sphenoid ridge. The meningioma exhibited numerous signal voids and dilated tortuous blood vessels, indicating high vascularity. Furthermore, they showed central high and peripheral low T2 signal intensity with avid homogeneous enhancement. Subsequent displacement and compression of ACA and MCA arteries and its branches. Also, a high focal T2/FLAIR signal with no diffusion restriction associated (Figure 1(d)).

Since 2015, the patient's visual acuity in both eyes was gradually decreasing. Furthermore, the patient lost the sense of smell and developed facial asymmetry. Currently, the visual acuity is limited to hand motion. Fundus examination revealed total bilateral optic atrophy in both eyes. Finally, the patient was admitted, and surgical resection of the anterior fossa tumor was planned. Angiography showed a hypervascular tumor supplied by numerous vessels. Because surgical resection was limited to the frontal component, we targeted our embolization to that territory. Subsequently, a 30%–40% decrease in vascularity was achieved. Right internal maxillary, right middle meningeal arteries and the left ophthalmic artery feeders were embolized. The remaining feeders were too many and too small to pick out individually. Those feeders were not attempted due to an unfavorable benefit-risk ratio. He had no postoperative neurological deficits. Histological examination of the specimen revealed atypical meningioma (WHO grade II) (Figures 2(e) and 2(f)). The tumor showed high cellularity and small cells with a high N/C ratio, and the foci of the pattern have less growth. The mitotic count reached 15 m/10 HPF in some foci. No necrosis, brain tissue, or sarcomatoid features were seen. Immunohistochemical stains showed a positive focal reaction for EMA and PR, and Ki67 was approximately 10%–15%.

## 3. Discussion

Meningioma is largely a benign indolent tumor originating from arachnoid caps [12]. Meningioma growth rate and pattern are linear and highly variable. Less than 40% demonstrating continues growth throughout 5 years while others discontinuing [1, 11]. It has a considerably high recurrence rate, and it may recur even 25 years after complete resection [13]. The location of the meningioma may influence how it behaves. For example, skull base meningioma tends to have a slower growth rate, and they are more benign in nature [14]. Up to 28.5% of the recurrent meningiomas were reported having malignant progression [15].

Multiple factors play a critical role in the decision-making for the management of meningiomas including tumor's size, location, and grade; patient's comorbidities; and history of prior resection [3, 4]. If feasible, upfront surgical resection is the management of choice for large and symptomatic masses. In contrast, the management of small benign asymptomatic meningiomas is controversial [3]. In the preradiosurgery era, observation with serial imaging until symptoms develop or growth confirmation sustained is frequently offered. Currently, prophylactic SRS is an effective alternative option to control tumor growth and symptoms associated [3, 16].

Stereotactic radiosurgery is usually recommended for small and moderate-size meningiomas with a maximum diameter of 3–3.5 cm [17]. Currently, the advocacy to use SRS for recurrent or residual tumors after initial surgical resection has been increasing. The combination of both treatment modalities is more effective, although not statistically significant [18]. However, other studies showed no difference. Moreover, progression has been inversely related to the early use of SRS after microsurgery in many series [18]. It was also advocated that SRS should be the initial treatment of choice if total removal is less likely [15]. In a study by Santacroce et al., they concluded that the risk for progression for subtotally removed meningiomas without further adjuvant therapy was as high as 70% [19]. Stereotactic radiosurgery is associated with early and delayed complications including alopecia, scalp paresthesia, dizziness, cognitive alteration, ataxia, cerebellar dysfunction, weakness, altered sensation, dysgraphia, Horner syndrome, seizures, hypopituitarism, perilesional edema, cranial nerves injury, infarction, secondary tumor formation, CSF leak, and death [5, 8, 10, 17, 20, 21]. These complications vary substantially according to tumor volume, location, and radiation dosage [12]. However, SRS has a low overall risk of complications [20].

The definition of tumor progression after SRS is inconsistent among previous studies. Progression can be defined as an over 15% increase of the original tumor volume and up to 20% in other studies [3, 14, 22, 23]. The control rate and progression-free survival after SRS are lower if there are a prior history of tumor progression after a surgical intervention or radiation therapy, lower radiation doses, larger meningioma size (>2.5 cm), presence of meningiomatosis, superficial meningiomas, higher grade, higher proliferative index (MIB-1 index > 3%), a lack of dural tail management, prolonged symptom duration, presence of cranial nerve deficit, male gender, and age over 65 years [1, 2, 4–7, 17–19, 24–27]. Over the years since the introduction of radiosurgery, the SRS-associated transformation (dedifferentiation) has been considered and described in several cases. The conversion after SRS in a histologically proven benign may not be necessary because of the radiation rather than because of the natural history of the disease. In several described cases, there were only a focus of histological changes surrounded by benign histology, but in other cases, the entire tumor showed complete dedifferentiation [11, 28]. The radiobiological basis between SRS and conventional radiotherapy is distinct. SRS modality is delivering a high dose of radiation in a very restricted area causing more cell death and fewer living cells with mutant DNA. Conversely, conventional radiotherapy delivers lower doses on a broader area causing fewer cell death and more cells with mutant DNA [29]. It was estimated that the risk of transformation and secondary tumor formation after SRS is between 0.04 and 2.6% in 15 years [7].

In this report, up to our knowledge, we reviewed all previous studies reporting patients diagnosed with grade I meningiomas histopathologically or presumed by radiological features of benign meningioma who underwent radiosurgery primarily or adjuvant. Studies that included patients with higher grades, genetic alterations, phakomatosis, and prior chemotherapy or EBRT were excluded. In a study by Park et al., 8 out of 200 patients had tumor progression after SRS requiring additional surgery at a median of 62 months. All eight patients who progressed had WHO grade I [26]. Likewise, in another recent study, 89 received SRS as primary or secondary, 5 showed progression, and only 3 of them required additional surgical decompression in a follow-up period of 75–164 months after SRS. All had WHO grade I [14]. Furthermore, in an extended study reporting 675 patients followed up to 252 months after SRS, they reported no transformation. In another study supporting the safety of SRS, there were 5000 patients with different intracranial tumors including meningioma of which >1200 patients followed for 10–19 years. Surprisingly, they found no risk of malignancy-related complications when it was compared to the general population [29]. By contrast, several studies reported treatment failure and higher-grade progression. In a study by Lee et al. which investigated the long-term outcome of SRS for the newly diagnosed small meningiomas as well as histopathological grading with a minimum follow-up period of one to 10 years after SRS, 9 out of 113 patients had tumor progression and 4 out of 9 were operated and results came as a transformation to grade II [3]. Similarly, Pollock and colleagues investigated the risk of malignant transformation as well as radiation-induced tumorigenesis after SRS, and they reported that 7 out of 316 meningioma patients demonstrated a higher-grade progression to grade II or III in a minimum follow-up of 5 years after SRS. All had proven a histopathological confirmation of grade I meningioma prior [7]. Also, they concluded that meningioma has a higher risk of transformation after SRS [7]. In a series presented by Couldwell et al., all benign tumors progressed after SRS. The meningiomas demonstrated extreme variability regarding the timing of progression ranging between early progression of 6 months after SRS and delayed growth 14 years after SRS. They concluded that tumors growing after failed SRS tend to be more aggressive and extended follow-up over ten years is recommended. In another nationally based study with 15 participating centers, they reported the long-term result for 3768 patients followed for at least five years after SRS; they had only eight malignant progressions [18]. Few case reports have previously described malignant progression. Of those, Kunert et al. presented a case of a 62-year-old woman with a small incidental benign-looking meningioma, which progressed slowly over 3 years and was managed initially with radiosurgery. After 3 months of radiosurgery, the patient developed progressive enlargement and an aggressive-looking tumor on MR. She underwent surgical removal, and results revealed transformation to WHO grade II [11]. In our case, a benign tumor was histopathologically confirmed before SRS and later radiological and histopathological progressions were observed. Other studies have demonstrated progression after SRS, but histopathological confirmation was lacking [2, 4, 10, 13, 18, 20, 22, 23, 27, 30–35]. A point to acknowledge is that some of the presented series might suffer from some (referral) bias such as the series presented by Couldwell et al.

Data in Table 1 seems to suggest that SRS has a considerably remarkably high rate for tumor control and that the risk of transformation after SRS is trivial. Thus, the use of SRS should not be precluded in medical practice. This view is supported previously by other authors [10]. Iwai et al. reported seven cases of transformation after SRS. They suggested no link between SRS and transformation rather than the natural course of the disease [15].

## 4. Conclusion

A case of meningioma transformation to a higher grade after SRS was described. The risk of progression after SRS is low, and the risk of transformation after SRS is trivial. Early timing for recurrence and field-related radiation may favor a relationship between SRS and transformation (causality) although transformation as part of the natural history of the disease cannot be entirely excluded. Tumor progression (treatment failure) after SRS may demonstrate grade progression. Careful, close, and prolonged follow-up is highly recommended. Acknowledging that there is a low risk of early and delayed complications and trivial risk of transformation should not preclude the use of SRS because it has a high level of safety and efficiency.

## Figures and Tables

**Table 1:** Outcome of benign meningioma patients who underwent stereotactic radiosurgery (SRS).

Study/publication year	No. of grade I meningioma patients treated with SRS as primary for presumed grade I or adjuvant after surgery	No. of patients who had radiological progression after SRS	Number of operated patients after SRS and progression	Reported histologic vs. presumed grade I meningioma before SRS	Histopathological confirmation after surgery	Grade progression to WHO grade II or III	Time to progression post-SRS. Mean or Median in months (Range)
Park et al. [26], 2018	200	28 (14%)	8	Mixed	Yes	No	62 (range 49-104)
M. R. Patibandla et al. [2], 2018	120	13 (10.8%)	2	Mixed	NR	Not reported	(52-84)
J. W. Kim et al. [14], 2017	89	5 (5.6%)	3	Mixed	Yes	No	(75-164)
Pollock et al. [7], 2017	316	7 (2.2%)	7	Histologic	Yes	Yes (7)	(33.6–165.6)
Kim et al. [24], 2016	865	56 (6.47%)	8^∗^	Presumed	Yes	Yes (6/8)	(36-228)
Lee et al. [3], 2016	113	9 (7.9%)	4 (3.5%)	Presumed	Yes	Yes (4)	49.4 ± 30.9 (24–120)
CK Jang et al. [1], 2015	628	28 (4%)	9	Mixed (128 has)	1 reported	Yes (1)	(1-79)
J. P. Sheehan et al. [5], 2015	675	59 (8.8%)	24	Mixed (292 has)	Yes	No	60.1 ± 45.4 (6–252)
R. M. Starke et al. [17], 2015	75	12 (16%)	5	Mixed (45 has)	Yes	No	78 (6–252)
C.J. Przybylowski et al. [18], 2014	34 (>WHO1 patients excluded)	5 (14.7%)	NR	Histologic	NR	NR	48.9 (1.6–61.4)
Seong-Hyun Park et al. [34], 2015	39	4 (10%)	0	Mixed (11 has)	—	—	57 (16–87)
R. Starke et al. [36], 2014	254	2-8	8	Mixed (114 has)	Yes	No	71.1 (6–252)
S. H. Park et al. [21], 2014	74	2 (3%)	1	Mixed (14 has)	NR	NR	At 14.6 and 17.1 after SRS, respectively
Pollock et al. [4], 2013	460	24 (5.7%)	NR	Mixed	NR	NR	(43-74)
B. E. Pollock et al. [6], 2013	115	6 (5%)	4	Mixed (46 has)	Yes	No	74 (42–145)
D. J. Salvetti et al. [23], 2013	42	1 (2.4%)	0	Mixed (19 has)	—	—	59 (12–144)
F.A. Zeiler et al. [37], 2012	30	2 (7.7%)	0	Mixed (12 has)	—	—	36.1 (3-80)
B. E. Pollock et al. [38], 2012	251	3 (1.2%)	0^∗^^2^	Presumed	—	—	At 28, 145, and 150 months, respectively
Santacroce et al. [19], 2012	3768	281 (7.5%)	27	Mixed	Yes	Yes (8/27)	Median, 48.7 Mean, 56
R. M. Starke et al. [27], 2011	152	19 (13%)	7	Mixed (77 has)	NR	NR	84 (24–192)
M. A. Dos Santos et al. [35], 2011	88	9 (10.2%)	0	Mixed (41 has)	—	—	86.8 (17.1–179.4)
K.-W. Jo et al. [10], 2011	69	0	0	Presumed	—	—	63.1 (24–110)
Zada et al. [39], 2010	136	7 (5.1%)	3	Mixed (72 has)	Yes	No	90 (60-122)
Skeie et al. [13], 2010	93	7 (7.5%)	7	Mixed (53 has)	NR	NR	82.0 ± 58.3 (1–240 mo)
H. Kuroda et al. [40], 2009	1	1	1	Histologic	Yes	Yes	(23-33)
P. Kunert et al. [11], 2009	1	1	1	Presumed	Yes	Yes	3 months after SRS
J.C. Ganz et al. [32], 2008	97	0	0	Presumed	—	—	54 (25–86)
Y. Iwai et al. [15], 2008	108	18 (16.7%)	NR	Mixed (62 has)	NR	Yes (7)	81.8 (20–144)
L. Davidson et al. [22], 2007	36	1 (2.8%)	1	Histologic	NR	NR	81 (30–141)
A. Kollová et al. [33], 2007	368	9 (2.5%)	5	Mixed (109 has)	NR	NR	60 (24-126)
W.T. Couldwell et al. [41], 2007	13	13	13	Mixed (10 has)	Yes	Yes (1)	(6-14)
Kubo O et al. [8], 2005	1	1	1	Histologic	Yes	Yes	—
S.J. Dibiase et al. [31], 2004	121	10 (8.3%)	1	Mixed (<52 has)	NR	NR	54 (3.96–126).
B.E. Pollock [42], 2003	267	6 (2.2%)	5	Mixed (107 has)	Yes	Yes (2)	43 (2–138)
Fuentes et al. [43], 2002	1	1	1	Histologic	Yes	Yes	(12-84)

∗: all patients treated by other than surgery after initial SRS were excluded. ∗2: ten patients operated for other reasons after SRS other than progression; all had WHO grade 1.NR : Not Reported.

## Data Availability

The data used to support the findings of this study are included within the article. Patient's hospital MRN number at PSMMC will be provided upon request after patient's and institutional approval.
